# Genetic Determinants of Microvascular Complications of Type 1 Diabetes Mellitus: New Data from a Replication Study

**DOI:** 10.3390/biomedicines14061199

**Published:** 2026-05-26

**Authors:** Bulat I. Yalaev, Rita I. Khusainova, Ildar R. Minniakhmetov, Dmitry D. Panteleev, Madina I. Yevloyeva, Minara S. Shamkhalova, Yulia Y. Golubkina, Ekaterina A. Dobreva, Marina V. Shestakova, Natalia G. Mokrysheva

**Affiliations:** Endocrinology Research Centre, Dmitry Ulyanov Street, 11, 117292 Moscow, Russia; khusainova.rita@endocrincentr.ru (R.I.K.); minniakhmetov.ildar@endocrincentr.ru (I.R.M.); panteleev.dmitry@endocrincentr.ru (D.D.P.); evloeva.madina@endocrincentr.ru (M.I.Y.); shamkhalova.minara@endocrincentr.ru (M.S.S.); golubkina.uliia@endocrincentr.ru (Y.Y.G.); dobreva.ekaterina@endocrincentr.ru (E.A.D.); shestakova.marina@endocrincentr.ru (M.V.S.); mokrisheva.natalia@endocrincentr.ru (N.G.M.)

**Keywords:** type 1 diabetes mellitus, SNP, retinopathy, nephropathy, complications, genetics

## Abstract

**Background**: Diabetic retinopathy (DR) and chronic kidney disease (CKD) are among the leading causes of disability in individuals with type 1 diabetes mellitus (T1DM). However, the genetic architecture of these complications remains poorly understood. Genome-wide studies demonstrate significant interpopulation heterogeneity, while candidate gene studies yield conflicting results due to limited power. Independent replication of previously obtained results in separate cohorts, with appropriate intergroup comparisons, is essential for identifying the most significant biomarkers of microvascular complications in T1DM. **Purpose**: To search for associations of the most significant polymorphic variants rs55703767, rs72831309, rs118124843, rs9344715, rs183937294, rs4293393, rs12917707, rs77924615, rs11864909, rs9622363, rs73885319, rs2523989, rs3825932, rs763361, rs12708716, rs2292239, and rs4948088 with the risk of developing T1DM and its complications—DR and CKD. **Methods**: The study involved 618 individuals, including 522 patients with T1DM undergoing inpatient treatment at the Endocrinology Research Centre, as well as 96 control individuals without T1DM. Among the T1DM patients, 232 had concurrent CKD and retinopathy, while 80 were free of both microvascular complications. A comparison of allele and genotype frequencies of 17 single-nucleotide polymorphisms (SNPs) was conducted between the T1DM group and the control group, as well as an intergroup comparison between individuals with and without complications. **Results**: The rs2292239 (*ERBB3*) locus is associated with an increased risk (p_bonf_ = 0.001, OR = 2.02), while rs55703767 (*COL4A3*) is associated with a decreased risk of developing T1DM in general (*p* = 0.01846, OR = 0.42). rs9344715 (*AKIRIN2*) is associated with the risk of diabetic nephropathy (*p* = 0.03996, OR = 1.29), while *PDILT* variants rs77924615 (OR = 0.57, p_bonf_ = 0.045) and rs11864909 (OR = 0.41, p_bonf_ = 0.0105) with DR. **Conclusions**: The study identified potential genetic markers for the risk of type 1 diabetes and its microvascular complications. The results require further verification in an independent, expanded cohort. Consideration of genetic factors confirmed the independent contribution of the identified variants, supporting their value as promising biomarkers for risk stratification and personalized prevention of T1DM complications.

## 1. Introduction

Type 1 diabetes mellitus is a polygenic, multifactorial disease caused by immune-mediated or idiopathic destruction of pancreatic β-cells. This chronic metabolic disorder leads to a variety of long-term microvascular complications, the most common of which are retinopathy, nephropathy, and neuropathy [1,2]. Currently, therapeutic approaches aimed at managing these complications face the problem of early diagnosis: by the time of clinical manifestation, young patients with diabetes usually already have vascular lesions that are resistant to modern treatment [3]. In children and adolescents with type 1 or type 2 diabetes mellitus, late diagnosis of microangiopathies is attributed to two factors: a prolonged asymptomatic course in the early stages of the disease and the multifactorial nature of the pathogenic mechanisms leading to complications. This, in turn, hinders the identification of specific early markers for screening purposes [4].

In Russia, the prevalence of chronic kidney disease among adult patients with diabetes showed a steady increase between 2010 and 2022. For type 1 diabetes, the rate increased from 21.5% to 27.1% (a 1.3-fold increase) [5]. The change in risk profiles for new cases of CKD deserves special attention. The proportion of patients with low and moderate combined risk of cardiovascular events and end-stage renal disease (ESRD) according to the KDIGO classification continues to increase. In type 1 diabetes, the proportion of this category of patients increased from 63.7% to 82.4%, consistent with the overall trend toward more frequent detection of complications characteristic of other microvascular pathologies, including diabetic retinopathy [5]. The prevalence of diabetic retinopathy among patients with diabetes mellitus exhibits significant spatial heterogeneity. This phenomenon is due to a combination of factors, including genetic characteristics of regional populations and differences in access to ophthalmological care. Based on extrapolation of data obtained from several studies, it is estimated that the total number of cases of diabetic retinopathy in Russia ranges from 767,000 to 1.5 million people. This alarming picture is compounded by a significant annual increase: the complication is estimated to be newly diagnosed in 48,000 to 62,500 patients per year [6].

It is known that chronic hyperglycemia stimulates excessive production of reactive oxygen species (ROS), which leads to disruption of the metabolism and structure of lipids, proteins, and DNA, as well as damage to the integrity of blood vessels [7]. An oxidative environment causes endothelial dysfunction by reducing nitric oxide bioavailability, promoting vasoconstriction, inflammation, and thrombosis. Activation of redox-sensitive transcription factors further enhances inflammation by increasing levels of adhesion molecules and cytokines, promoting leukocyte infiltration into the endothelium, and accelerating vascular complications in the heart, kidneys, and eyes [7]. Despite the understanding of these mechanisms, the clinical heterogeneity of chronic kidney disease and ophthalmopathy remains a major unresolved issue. A study conducted at the Joslin Diabetes Center, which examined the histories of patients with T1D for over 50 years, found that 30–35% of them do not have significant microvascular complications, regardless of glycated hemoglobin (HbA1c) levels and other classic risk factors thought to predict nephropathy and retinopathy [8]. This indicates the inadequacy of analyzing the dynamics of metabolic factors for early risk prediction. The need to identify reproducible genetic markers associated with resistance to microvascular complications or a predisposition to their progression naturally comes to the fore.

Large-scale GWAS studies of kidney function in both non-diabetic individuals and patients with T1D and T2D have identified a number of genetic markers of high statistical significance, which, at the same time, vary between different studies. For example, in the work of Khattab et al. (2022), as part of a genome-wide association study (GWAS), the most significant signal was found for variants of the *UMOD* and *PDILT* genes [9]. The *UMOD* gene encodes uromodulin, which is involved in protecting the kidneys from infections and salt crystallization; *PDILT* is a protein in the endoplasmic reticulum disulfide isomerase family, which catalyzes protein folding and thiol-disulfide exchange reactions [10]. Another study conducted by the Diabetic Nephropathy Collaborative Research Initiative (DNCRI) identified 16 significant genome-wide variants in a sample of 19,000 people with type 1 diabetes. Notably, one of the strongest associations was with a protective variant in the *COL4A3* gene. The authors interpreted the effect of the risk variant in *COL4A3* as being associated with thinning of the glomerular basement membrane only in the presence of concomitant hyperglycemia (HbA_1_c > 58 mmol/mol), indicating an important role for the relationship between collagen gene variation and metabolic background in type 1-associated microvascular complications [11]. The results of an extensive meta-analysis (N = 27,000; T1D and T2D groups) are noteworthy. This study, for the first time, identified an association between the intronic variant rs72831309 of the *TENM2* gene (transmembrane protein teneurin 2) and a reduced risk of developing comorbid forms of chronic kidney disease. In the same study, the integration of GWAS results with gene expression data in human kidneys revealed higher expression of the *AKIRIN2* gene in the tubules of individuals with diabetic kidney disease [12]. Teneurin 2 mediates the binding of cell adhesion molecules and signaling receptors [13], and AKIRIN2 is involved in proteasome localization and protein import into the nucleus, playing an important role in cellular processes [14].

Recent genome-wide studies have linked the rs1024611 variant in the *MCP-1* (monocyte chemotactic protein-1) gene and the rs3025039 variant in the *VEGF* (vascular endothelial growth factor) gene with diabetic retinopathy in type 2 diabetes. Variants such as rs2268388 in the *ACACB* gene, insertions/deletions in the *ACE* (angiotensin-converting enzyme type I) gene, rs1801133 in the *MTHFR* (methylenetetrahydrofolate reductase) gene, and rs7903146 in the *TCF7L2* gene are also associated with diabetic kidney disease in type 2 diabetes. Furthermore, rs4880 in the *SOD2* (superoxide dismutase 2) gene has been associated with diabetic peripheral neuropathy in type 1 diabetes [15].

In addition, two key polymorphisms with opposite effects on diabetic retinopathy (DR) were identified: the rs3825932 variant in the *CTSH* (cathepsin H) gene is associated with a reduced risk, likely through modulation of the immune response and β-cell homeostasis, while the rs2292239 variant in the *ERBB3* (HER3 receptor) gene may contribute to the development of vascular complications through effects on angiogenesis and the blood-retinal barrier [16,17]. Genes associated with immune-inflammatory damage (*TRIM31* [18], *CD226* [19], *CLEC16A* [20]) and cellular vulnerability to hyperglycemia (*LRP5*) [21] are also in focus and are associated with the risk of T1DM. However, the available data, including the results of genome-wide searches, are characterized by low reproducibility, which is often due to methodological differences.

It is becoming clear that a comprehensive study of the nature of microvascular complications in type 1 diabetes is impossible without a deep, integrated analysis linking genetic predisposition to known pathogenetic mechanisms. These genes regulate key components of the immune response, autophagy, and cellular survival—processes that, while initially involved in autoimmune destruction of β-cells, may similarly modulate chronic inflammatory damage to the retinal vascular endothelium and renal glomeruli. Based on these considerations, the need for replication studies of polymorphic variants of these genes in an intergroup comparison of individuals with uncomplicated diabetes, diabetes with diabetic retinopathy, and diabetes with chronic kidney disease is particularly urgent. This is not only advisable but also a methodologically necessary step in verifying their clinical and pathogenetic significance, including from the perspective of studying the influence of gene variability on the risk of developing combined forms of type 1 diabetes complications. Although some genes (such as *CTSH*, *CD226*, *CLEC16A*, and *ERBB3*) were initially associated only with the risk of developing T1DM itself, their potential contribution to the pathogenesis of complications remains hypothetical and requires additional research on independent samples accounting for different factors.

This study aims to investigate associations of the following polymorphic variants with type 1 diabetes mellitus overall, as well as with diabetic retinopathy and nephropathy in men and women from the Russian Federation: rs2523989 (*TRIM31*), rs3825932 (*CTSH*), rs763361 (*CD226*), rs12708716 (*CLEC16A*), rs2292239 (*ERBB3*), rs4948088 (*COBL*), rs55703767 (*COL4A3*), rs72831309 (*TENM2*), rs118124843 (*VARS2*), rs9344715 (*AKIRIN2*), rs183937294 (*PLEKHA7*), rs4293393 (*UMOD*), rs12917707 (*UMOD*), rs77924615 (*PDILT*), rs11864909 (*PDILT*), rs9622363 (*APOL1*), and rs73885319 (*APOL1*).

## 2. Materials and Methods

### 2.1. Participants

**Figure 1:**
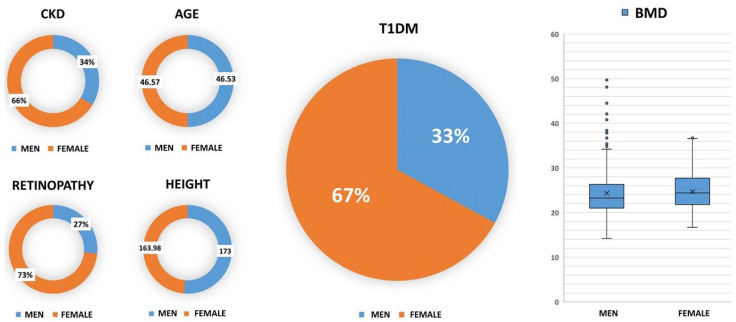
Diagrammatic representation of summary statistics of sample characteristics.

The study involved 618 individuals, including 522 patients with type 1 diabetes mellitus (mean age 44.51, standard deviation 11.75) undergoing inpatient observation at the Endocrinology Research Centre, as well as 96 individuals from the control sample without a history of type 1 diabetes (mean age 57.69, standard deviation 11.55). Among patients with type 1 diabetes, 241 had CKD, and 433 had diabetic retinopathy; these groups overlapped, with 232 patients experiencing both complications simultaneously (Table 1 and Figure 1). The sample also included 80 patients with type 1 diabetes who lacked both CKD and DR. Overall, the mean duration of type 1 diabetes mellitus in the sample was 30 years at the start of the study. A total of 618 individuals in the group included 390 women (63.11%) and 228 men (36.89%). All participants were of Russian ethnicity (100%). Ethnicity was determined based on questionnaire data, based on the origin of ancestors in three generations (parents and grandparents). The median age of disease onset in men was 12 years (standard deviation ± 9 years) and in women, 11 years (standard deviation ± 8.75 years). The overall median age at T1DM onset for the entire sample was 11 years (standard deviation ± 8.3 years). Baseline characteristics of the study sample are summarized in Table 1.

Ethical approval was obtained from the Ethics Committee of the Endocrinology Research Centre in accordance with Protocol No. 16 dated 13 September 2023. All participants or their legal representatives provided written informed consent in accordance with the Declaration of Helsinki. The consent form detailed the purpose of the study, the procedures used, the risks, and the confidentiality of personal data.

Inclusion criteria included individuals with type 1 diabetes mellitus, either without chronic kidney disease and retinopathy, or with chronic kidney disease and retinopathy, or with a combination of both at different stages, as well as the mandatory presence of signed informed consent from patients to participate in the study. Age, sex, duration and onset of type 1 diabetes, anthropometric parameters (BMI), and renal function data (eGFR, albuminuria, albumin-to-creatinine ratio) were recorded for all participants. The diagnosis of diabetic retinopathy was established based on a standardized ophthalmological examination (direct and indirect ophthalmoscopy, biomicroscopy).

The control group consisted of healthy participants undergoing routine preventive medical examinations. Exclusion criteria were: any current or past diagnosis of diabetes, impaired glucose metabolism, autoimmune disease, malignancy, or chronic condition requiring long-term pharmacotherapy (hypertension, coronary artery disease, CKD, osteoporosis); any current or past hormonal therapy; and a family history of type 1 diabetes or autoimmune diseases in first- or second-degree relatives.

Total exclusion criteria included the presence of oncological diseases, acute myocardial infarction or acute cerebrovascular accident within 12 weeks prior to inclusion, pregnancy and lactation, alcohol or drug addiction, as well as the absence of up-to-date anamnestic data and laboratory and instrumental research results for 2022–2024.

### 2.2. Criteria for Choosing Loci for Analysis

**Figure 2:**
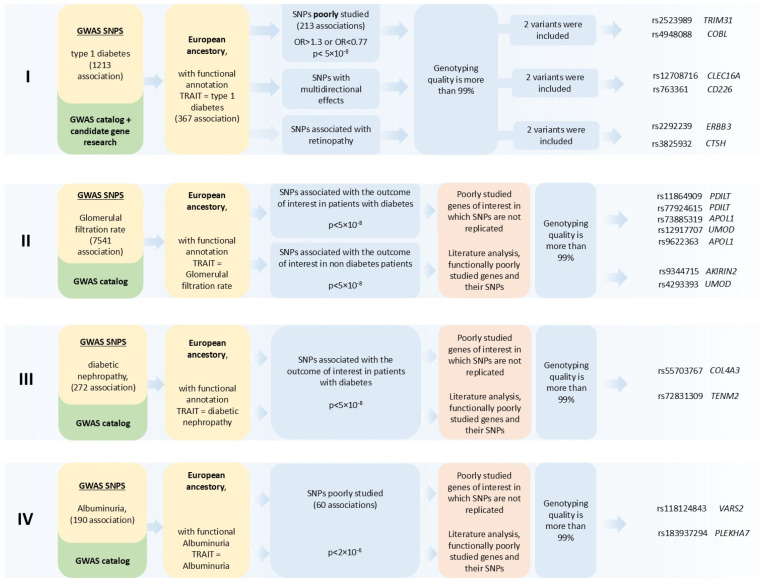
Design selection of polymorphic variants.

**Figure 3:**
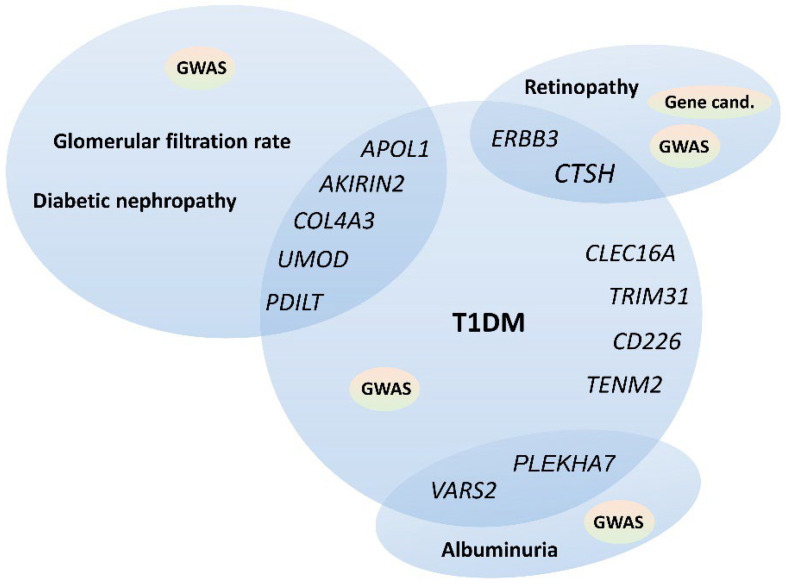
Venn plot of the distribution of genes according to the studied traits.

The selection of 17 single-nucleotide variants for replication analysis was performed based on predefined criteria aimed at testing hypotheses about genetic predisposition to microvascular complications of type 1 diabetes (Figure 2 and Figure 3).

The selection strategy included: (1) prioritization of variants previously shown to be associated with type 1 diabetes mellitus, diabetic retinopathy, chronic kidney disease, or related quantitative traits (albuminuria, glomerular filtration rate) in genome-wide association studies (GWAS); (2) focusing on loci that have not been adequately studied in the context of their contribution to the pathogenesis of microvascular complications in type 1 diabetes, particularly in the Russian patient population; and (3) selection based on the biological role of genes in immune regulation, kidney function, extracellular matrix integrity, and intracellular signaling.

The table below summarizes the literature-based rationale for including each of the 17 SNPs in the analysis (Table 2). The presented flowchart (Figure 2) and Venn diagram (Figure 3) reflect a multi-step strategy for selecting loci for a replication study integrating five interrelated phenotypes: type 1 diabetes mellitus, retinopathy, diabetic nephropathy, glomerular filtration rate, and albuminuria.

### 2.3. Isolation of Genomic DNA and Genotyping of Polymorphic Variants

Genomic DNA was isolated from peripheral blood lymphocytes using the MagPure Blood DNA kit (Magen, Guangzhou, China). The quantity and purity of the isolated DNA were assessed using a Nanodrop 2000 spectrophotometer (Thermo Fisher Scientific, Waltham, MA, USA) and a Qubit 2.0 fluorometer (Invitrogen, Carlsbad, CA, USA) with the Qubit dsDNA HS Assay Kit.

Genotyping of the studied samples was performed using TaqMan real-time polymerase chain reaction technology on a QuantStudio 5 platform (Thermo Fisher Scientific, Waltham, MA, USA). The selection of genetic loci for the study was based on genome-wide association study data. When selecting loci from GWAS, the level of statistical significance (*p* < 5 × 10^−8^) was taken into account. The study included loci that demonstrated a consistent association with diabetic microvascular complications (specifically, diabetic nephropathy and retinopathy) in large meta-analyses and genome-wide association studies, as described in the article’s literature review. The characteristics of the studied polymorphic variants are presented in Table 3.

### 2.4. Statistical Analysis and Interpretation

Differences in DM duration between groups were assessed using both the parametric Student’s *t*-test and the non-parametric Mann–Whitney U test (the latter applied due to slight deviations from normality as indicated by the Shapiro–Wilk test, *p* < 0.05 in certain subgroups). For each polymorphic variant, an association test was performed using Pearson’s chi-squared test to compare genotype and allele frequencies between groups (e.g., patients with complications versus those without complications; patients with T1DM versus healthy controls). The test was applied directly to contingency tables (allelic 2 × 2, genotypic 2 × 3). PLINK software (v. 1.09) was used to search for associations of alleles and genotypes with diabetes in case–control analyses. Logistic regression models were employed to further evaluate the associations of polymorphic variants with the studied phenotypes, adjusting for additional factors.

Binary logistic regression was used to develop predictive models for T1DM and its microvascular complications. For the model comparing T1DM patients with controls, the initial set of candidate predictors included age, sex, and all 17 analyzed SNPs. For models of microvascular complications (diabetic retinopathy and chronic kidney disease) within the T1DM group, T1DM duration was additionally included as a candidate predictor. Genotypes were coded as categorical variables using the most frequent homozygous genotype in the control group as the reference level. Variable selection was performed using backward stepwise logistic regression based on the likelihood ratio test (entry threshold *p* < 0.05, removal threshold *p* > 0.10). Prior to final model fitting, multicollinearity was assessed (variance inflation factor, VIF < 2 for all retained predictors), and linearity of the logit was verified for continuous covariates. Model performance was evaluated using two components: discrimination (area under the ROC curve, AUC, sensitivity and specificity at the Youden index) and calibration (Hosmer–Lemeshow test, Brier score, calibration slope and intercept). Statistical significance was set at *p* < 0.05. Sex was included as a candidate covariate in all initial multivariable models. Predictors were retained in the final model only if they met the pre-specified stepwise selection threshold (*p* < 0.05 for retention). For the in silico assessment of the functional significance of intronic polymorphic variants that showed a statistically significant association with the studied traits, data from the RegulomeDB and MobiDB web platforms were used; for missense variants, the MoBiDiC MPA algorithm was applied [31].

## 3. Results

### 3.1. Analysis of Hardy–Weinberg Equilibrium

Hardy–Weinberg equilibrium analysis showed that the vast majority of the studied polymorphic variants corresponded to the expected genotype distribution both in the overall sample and in the control and T1DM groups as a whole (Table 4).

### 3.2. Analysis of Associations with Diabetes and Its Duration in General

A comprehensive analysis was conducted to examine the association between the duration of type 1 diabetes mellitus (DM duration, years) and the development of two major microvascular complications—chronic kidney disease (CKD) and diabetic retinopathy. For both retinopathy and CKD, a statistically significant positive association with disease duration was observed (*p* < 0.005 according to both tests). Multivariate analysis was performed using binary logistic regression (model: complication ~ DM duration). The results confirm an independent association between DM duration and the risk of developing retinopathy as well as the composite outcome (presence of either complication). A complete analysis was carried out for all 17 single-nucleotide polymorphisms (SNPs) included in the study. Univariate analysis (ANOVA/Kruskal–Wallis test) revealed no statistically significant differences in DM duration across genotypes for any of the examined SNPs (all *p* > 0.05). Interaction testing within the logistic regression framework (model: complication ~DM duration × genotype) demonstrated the absence of a modifying effect for all studied SNPs (all interaction *p*-values > 0.10).

Stratified analysis by individual genotype further confirmed that the significant association between DM duration and both retinopathy and CKD persisted in every genotypic subgroup (*p* < 0.01 for each comparison). Thus, the examined genetic variants neither influence disease duration nor modify its relationship with the risk of microvascular complications. The results demonstrate a statistically significant positive association between the duration of type 1 diabetes mellitus and the development of diabetic retinopathy, CKD, and their combined endpoint. Genetic polymorphisms do not act as modifiers of this effect. These findings underscore the importance of accounting for cumulative hyperglycemic exposure in risk models for microvascular complications and are consistent with the well-established pathophysiological role of disease duration.

The study initially conducted an association analysis of polymorphic genetic variants with type 1 diabetes mellitus as a whole, without taking into account the presence of microvascular complications, to establish whether the studied loci are associated with the disease itself, independent of the development of concomitant pathological conditions (nephropathy and retinopathy). The analysis was performed on the full sample of patients with verified T1DM and a control group of conditionally healthy individuals without T1DM. For each polymorphic locus, allele and genotype frequencies were calculated, after which the statistical significance of differences between the groups was assessed using the chi-square test and logistic regression adjusted for potential confounders (age, sex). The level of statistical significance was set at *p* < 0.05; the Bonferroni correction was used for multiple comparisons (Table 5).

Association analysis using the Pearson χ^2^ criterion revealed a significant association of the T allele of the rs2292239 polymorphic variant of the *ERBB3* gene with type 1 diabetes mellitus, which is confirmed by a significant difference in its frequency between the case group (0.389 for T1DM) and the control group (0.240), a high value of the χ^2^ statistic = 15.59, reaching the level of statistical significance after Bonferroni correction (*p* = 7.89 × 10^−5^, p_bonf_ = 0.001341), as well as an OR = 2.02 with a 95% confidence interval from 1.418 to 2.878 (Table 5).

Analysis of genotype distributions between the case and control groups using the overall Pearson goodness-of-fit test revealed a statistically significant difference (χ^2^ = 16.01, df = 2, *p* = 0.00033). When testing the recessive inheritance model (T/T genotype vs. the combined group of T/G and G/G carriers), the T/T homozygote was associated with an elevated risk of T1DM: the frequency of the T/T genotype was 15.3% (80/522) in cases and 6.25% (6/96) in controls (χ^2^ = 5.58, OR = 2.71, 95% CI 1.15–6.42, *p* = 0.018). This association did not remain significant after Bonferroni correction for multiple comparisons (p_bonf_ = 0.306) and is therefore considered nominal. These findings are fully concordant with the allele-based analysis: both the T allele and the T/T genotype consistently confer an increased risk of type 1 diabetes (Table 6). As a result of the genotype distribution analysis of the rs55703767 locus of the *COL4A3* gene between the T1DM group (N = 522) and the control group without T1DM (N = 96), it was found that the homozygous T/T genotype is associated with a reduced risk of developing T1DM within the framework of the recessive inheritance model (T/T vs. T/G + G/G) (χ^2^ = 5.56, OR = 0.42, 95% CI: 0.20–0.88, *p* = 0.018). However, after correction for multiple comparisons, the result is at the border of statistical significance (p_bonf_ = 0.05).

The search for associations of alleles depending on gender revealed that in men, in the same comparative analysis there is a tendency for the *T* allele of the rs2292239 variant of the *ERBB3* gene to be associated with type 1 diabetes (*p* = 0.05016), while in women the association remains (*p* = 0.002369), which is most likely explained by the larger sample size of women. Similarly, a search for associations of genotypes depending on gender was conducted, which revealed the preservation of associations in both genders (*p* < 0.05). Thus, a stratified analysis of the associations of the rs2292239 variant of the *ERBB3* gene with T1DM did not reveal significant gender differences, since a significant association was confirmed in the group of women, while in men it was of borderline significance, which is probably due to the smaller size of the male subgroup.

**Figure 4:**
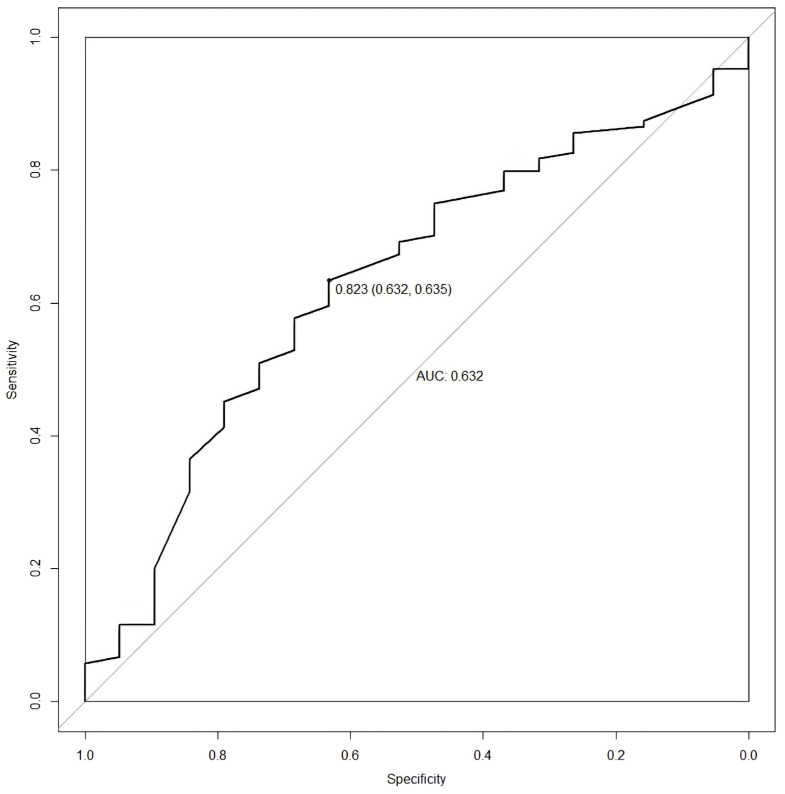
ROC curve of the analysis of the prognostic model of type 1 diabetes, including predictors rs55703767_T/T, rs2292239_G/T and rs2292239_T/T.

**Figure 5:**
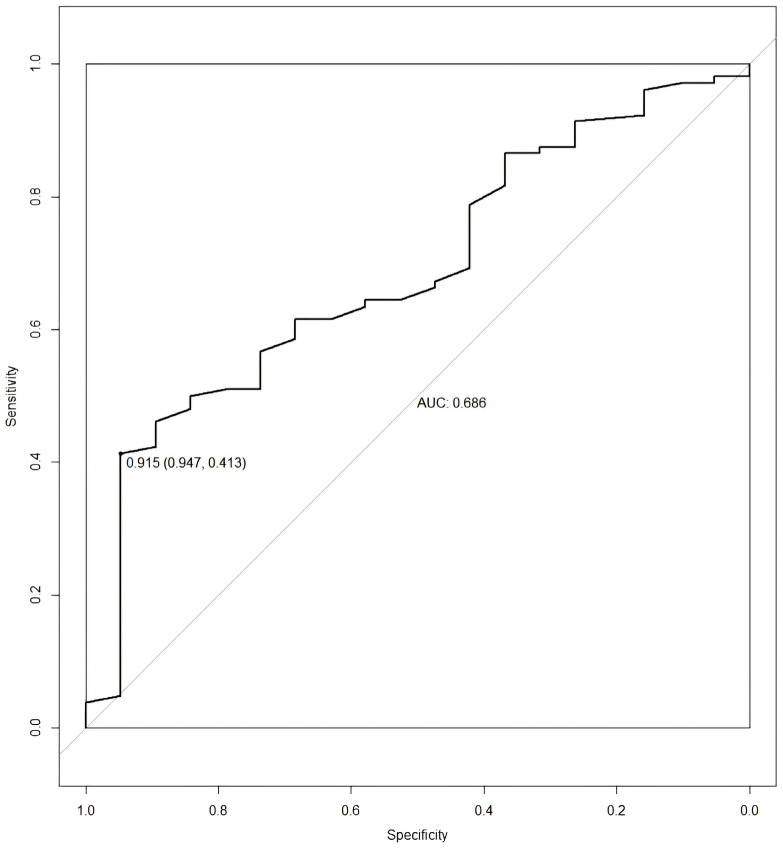
ROC curve of the analysis of the prognostic model of T1DM, including the predictors of the male sex factor, rs55703767_TT, rs2292239_GT and rs2292239_TT.

Stepwise selection identified independent predictors of T1DM risk: the T/T genotype of rs55703767 (*COL4A3*), the G/T and T/T genotypes of rs2292239 (*ERBB3*), and male sex. The T/T genotype of rs55703767 was associated with a significantly lower risk of T1DM. Age was excluded during variable selection (*p* > 0.10). Full regression coefficients, standard errors, 95% confidence intervals, *p*-values, and odds ratios are presented in Table 7. The model containing only genetic variants demonstrated moderate discriminatory ability (AUC = 0.632; sensitivity = 0.635; specificity = 0.632; Figure 4). Inclusion of sex improved model performance (AUC = 0.686; sensitivity = [0.413]; specificity = [0.947]; Figure 5). Calibration of the final model showed adequate agreement between predicted probabilities and observed outcomes: Hosmer-Lemeshow χ^2^ = 7.84, df = 8, *p* = 0.449; Brier score = 0.198; calibration slope = 0.94; intercept = 0.06. Male sex was associated with reduced odds of T1DM (OR = 0.33), whereas the T/T genotype of rs2292239 increased risk nearly fourfold compared with the reference genotype. When diabetic retinopathy and chronic kidney disease were used as dependent variables, no statistically significant associations were observed (*p* > 0.05 for all predictors); likely due to quasi-complete separation given the limited number of events for some rare variants. Logistic regression analysis with diabetic retinopathy (DR) and chronic kidney disease (CKD) as dependent variables revealed no statistically significant associations (*p* > 0.05) for the studied loci. The lack of significant models may be due to insufficient statistical power, the complex genetic architecture of microvascular complications, or the influence of unaccounted clinical and environmental factors. We note that at no stage of the analysis—neither when using the Pearson test nor in regression models without adjusting for disease duration—were any associations detected between the studied SNPs and T1DM duration. Although sex was evaluated alongside all 17 SNPs in the initial candidate set, it was retained in the final parsimonious model only together with rs2292239 and rs55703767 after stepwise selection, reflecting its independent contribution to T1DM risk prediction.

Thus, the analysis confirmed the association of two genetic loci with the risk of developing type 1 diabetes overall when compared with a control group of individuals without diabetes. The rs2292239 variant of the *ERBB3* gene and the rs55703767 variant of the *COL4A3* gene were shown to have opposite effects on the risk of developing T1DM.

### 3.3. Analysis of Polymorphic Variant Associations with Diabetic Nephropathy

After analyzing the associations of polymorphic variants with T1DM in general, the study focused on identifying specific genetic determinants of individual microvascular complications. Associations with chronic kidney disease were analyzed primarily, given its key clinical significance and direct pathophysiological link to several genes under study, such as *UMOD* and *APOL1*. The results of a comparison between a group of patients with uncomplicated T1MD and a group with established CKD are presented below to test the hypothesis of a statistically significant association between the studied variants and the risk of developing this complication.

When analyzing the frequencies of alleles and genotypes in a group of individuals with chronic kidney disease (diabetic nephropathy) compared with a group of individuals with diabetes mellitus but without CKD, it was found that the A allele of the polymorphic variant rs9344715 of the *AKIRIN2* gene is associated with this complication (χ^2^ = 4.220, OR = 1.29, CI = 1.012–1.648, *p* = 0.03996, p_bonf_ = 0.559), but does not pass the correction for multiple comparisons (Table 8).

As a result of the analysis of the genotype distribution of the rs9344715 variant between the group with and without chronic kidney disease, no statistically significant differences were found using the general Pearson goodness-of-fit test for the three genotypes (χ^2^ = 5.67, *p* = 0.059). However, when testing the recessive inheritance model for the A allele, comparing the homozygous genotype *A*/*A* with the combined group of carriers of the *A*/*G* and *G*/*G* genotypes, an association of the A/A genotype with an increased risk of nephropathy in type 1 diabetes was revealed (χ^2^ = 5.310, OR = 1.65, 95% CI: 1.075–2.531, *p* = 0.021), while the level of significance is not retained after Bonferroni correction for multiple comparisons (p_bonf_ = 0.064) (Table 9).

The next step involved constructing logistic regression models to identify significant predictors of nephropathy in type 1 diabetes among the studied loci. The models were evaluated using the inclusion and exclusion method for different loci.

Initially, all variants were included in the model. The G/A genotype of the rs9344715 polymorphism was found to be a significant protective factor reducing the risk of developing nephropathy in T1DM (B = −0.7145, *p* = 0.0467). However, ROC analysis revealed a low area under the curve (AUC = 0.523), indicating relatively low predictive power of the model. It is likely that with a larger sample and increased statistical power, these performance metrics would improve.

### 3.4. Analysis of Polymorphic Variant Associations with Diabetic Retinopathy

Following the analysis of associations between polymorphic variants and type 1 diabetes mellitus and diabetic nephropathy, the study shifted its focus to assessing their relationship with the second key microvascular complication: diabetic retinopathy. This phase aimed to identify specific genetic determinants associated with retinal vascular damage. Focusing on retinopathy allowed us to determine whether the loci under study, particularly those associated with angiogenesis and blood-retinal barrier integrity, have a selective effect on this phenotype.

Association analysis using the Pearson χ^2^ criterion revealed two risk polymorphic loci significantly associated with the development of diabetic retinopathy. However, this applies only to major alleles and genotypes. The analysis revealed that for the rs77924615 variant of the *PDILT* gene, a more frequent allele *G* is associated with an increased risk (χ^2^ = 6.160, OR = 1.59; 95% CI: 1.100–2.292; *p* = 0.01307), and for the rs11864909 variant of the *PDILT* gene, the major allele C is also associated with an increased risk (χ^2^ = 5.923, OR = 1.52; 95% CI: 1.083–2.131; *p* = 0.015). The obtained data indicate a significant role for these genetic markers in the risk of developing diabetic retinopathy (Table 10). However, the associations do not persist after Bonferroni correction for multiple comparisons (p_bonf_ = 0.1829 and p_bonf_ = 0.1943, respectively).

Analysis of the genotype distribution at the rs77924615 locus (*A*/*A*, *A*/*G*, *G*/*G* genotypes) of the gene between the group of patients with diabetic retinopathy (DR, N = 433) and the group with T1DM without retinopathy (N = 89) using the Pearson χ^2^ criterion revealed a statistically significant association of the *A*/*A* genotype with a reduced risk of developing retinopathy compared with *G*/*G* homozygotes (within the dominant model) (OR = 0.57, 95% CI: 0.36–0.90, χ^2^ = 5.815, *p* = 0.015) (Table 11). After Bonferroni correction, the adjusted p_bonf_ was 0.045.

As a result of the analysis of the genotype distribution of the rs11864909 locus of the *PDILT* gene between the group of patients with diabetic retinopathy (N = 433) and the group of patients with type 1 diabetes without retinopathy (N = 89) using the Pearson χ^2^ method, it was found that the *T*/*T* genotype (*T*/*T* vs. *T*/*C* + *C*/*C*) is associated with a reduced risk of developing diabetic retinopathy within the framework of the recessive inheritance model for the T allele (χ^2^ = 8.52, OR = 0.41, 95% CI: 0.225–0.759, *p* = 0.003) after the Bonferroni correction for the three tested models, the adjusted significance level was p_bonf_ = 0.0105) (Table 12).

The next step was to construct logistic regression models to search for significant predictors of diabetic retinopathy in type 1 diabetes among the studied loci, which, however, did not lead to any significant results (*p* > 0.05).

It is worth noting that the polymorphic variants rs183937294, rs9622363, and rs73885319 in the analyzed sample demonstrate homozygosity for the predominant allele due to the extremely low frequency of the alternative (minor) allele in populations of European origin, which is consistent with data from global genomic frequency databases. According to Ensembl, the minor allele G for rs183937294 has an MAF < 1% in the European subset of 1000 Genomes, with zero frequency in a number of subcohorts; for rs9622363, the MAF in the European population is 0%; similarly, for rs73885319, the MAF of G is <0.001 in European gnomAD v4 exomes.

In contrast, the analysis confirmed the association of two other genetic loci with the risk of developing diabetic retinopathy in type 1 diabetes mellitus. The common variants rs11864909 and rs77924615 (both in the *PDILT* gene) were shown to exert a risk effect on the development of this complication.

### 3.5. Analysis of Polymorphic Variant Associations with a Combined Form of T1DM Complications

The study also included an association analysis aimed at comparing the distribution of alleles and genotypes between a group of individuals with chronic kidney disease comorbid with retinopathy in type 1 diabetes and a group with type 1 diabetes without complications. The search for associations revealed a statistically significant association of the C allele of the rs11864909 polymorphic variant of the *PDILT* gene with the combined form of CKD and DR (χ^2^ = 4.348, OR = 1.49, 95% CI: 1.023–2.179, *p* = 0.037) (Table 13). Analysis of the genotype distribution of the rs11864909 locus between the group with CKD and DR (N = 232) and the group without complications (N = 80) revealed a statistically significant association of the homozygous T/T genotype with a reduced risk of developing the combined form within the recessive inheritance model for the T allele (T/T vs. T/C + C/C): χ^2^ = 6.01, OR = 0.44, 95% CI: 0.23–0.86, *p* = 0.014). After Bonferroni correction for the three tested models, the adjusted significance level was p_bonf_ = 0.043. No significant associations were found in the dominant (T/T + T/C vs. C/C) or additive models (Table 14). Thus, the protective effect of the rs11864909 locus with respect to the combined condition is manifested exclusively in the recessive inheritance model: the T/T genotype is associated with a reduced risk, which remains significant after Bonferroni correction (p_bonf_ = 0.043).

The absence of significant associations in other models indicates that the protective effect is specific to the homozygous state of the T allele, suggesting that two copies of the allele are required for the observed protective effect.

### 3.6. In Silico Functional Annotation of Prioritized Variants

Polymorphic variants rs2292239 (*ERBB3*: c.875-147T>G), rs77924615 (*PDILT*: c.409 + 3635C>T), and rs11864909 (PDILT: c.203-4666G>A) are located in introns; variant rs9344715 (AKIRIN2<>LOC101928911: n.87738792A>G) is located in an intergenic region (associated with an uncharacterized lncRNA gene), while rs55703767 (*COL4A3*: c.976G>A) is a missense substitution. Table 15 shows the association of non-coding variants with transcription factors (TF) and their motifs according to the Regulome DB database, and also presents the prioritization data for the missense variant rs55703767 obtained using the MPA MoBiDiC tool [31].

Functional annotation databases indicate that the intronic variant rs2292239 has regulatory potential mediated through binding to key transcription factors (CTCF, POLR2A, RBFOX2, CEBPA) and altered chromatin accessibility (Table 14). caQTL (chromatin accessibility QTL) and eQTL data confirm that this allele potentially influences local regulatory activity, likely by disrupting the binding sites of these transcription factors and affecting gene expression through altered chromatin accessibility in regulatory elements. However, further confirmation based on functional studies is required.

It is worth noting that the rs2292239 variant has no predicted effect on splicing and is classified as a benign variant in pathogenicity databases, which further limits our understanding of its possible molecular mechanisms and points to its specific role in transcriptional regulation. The annotation of the rs77924615 variant, located in the regulatory region upstream of the *PDILT* gene, indicates its potential role in transcriptional modulation. The locus is located in a kidney cis-regulatory element (cCRE) and is thought to alter chromatin accessibility in tubular epithelial cells by disrupting binding sites of several transcription factors [21]. These include key regulators of organ development, such as members of the nuclear factor I family (NFIA, NFIB, NFIC, NFIX), as well as tissue-specific factors HNF1A and CEBPA. The association of rs77924615 with serum uromodulin levels, glomerular filtration rate, and the risk of chronic kidney disease is consistent with its putative regulatory function in the nephron [32]. According to SpliceAI, the allelic status of rs77924615 does not affect the splicing process.

Based on RegulomeDB data, the rs9344715 variant was assigned a score of 1f, indicating high regulatory potential due to eQTL/caQTL associations at chromatin accessibility peaks. ChIP-seq data confirmed the binding of transcription factors, including ATF7 (a chromatin stress-responsive regulator), MTA2 (a component of the NuRD complex involved in gene repression), IKZF1 (a regulator of hematopoietic lymphoid cells), DPF2 (a subunit of the BAF complex mediating myeloid differentiation), MLLT1 (a component of the SEC complex for transcriptional elongation), and TBX21 (a master regulator of IFN-γ expression in Th1 lymphocytes). Variant rs55703767 (p.Asp326Tyr), which involves the replacement of aspartic acid with tyrosine at position 326 of the protein chain, is a likely benign variant with a bias toward unknown clinical significance (Franklin ACMG Classification). The variant is classified as a potentially pathogenic substitution in the genome (CADD Phred = 19.99). A high FATHMM-MKL score (converted rank score = 0.938) indicates a putative negative impact on regulatory elements. However, SpliceAI data (maximum delta score = 0.31 for acceptor site loss) together with a low integrated MPA indicator (final score = 2, “Low impact”) indicate the absence of a significant effect on the splicing process. Thus, the molecular mechanism of action of this variant is likely associated with transcriptional dysregulation (Table 16). These data are purely predictive and require confirmation in independent experimental systems.

## 4. Discussion

It is important to emphasize the importance of distinguishing the specific development of the pathogenetic processes of diabetic nephropathy and retinopathy in individuals with type 1 and type 2 diabetes. In type 1 diabetes, microvascular complications, particularly retinopathy and nephropathy, develop primarily under the influence of hyperglycemia without the significant contribution of concomitant metabolic factors (arterial hypertension, obesity, dyslipidemia) characteristic of type 2 diabetes [33,34]. For this reason, target organ damage in type 1 diabetes is characterized by more stringent pathogenetic specificity: its key mechanisms, such as protein glycation, oxidative stress, and intraglomerular hypertension, manifest themselves with minimal interference from external factors [35]. This pathogenetic homogeneity makes type 1 diabetes an optimal model for studying the genetic determinants of complications: a reduction in the number of confounding factors increases the reliability of associations between genetic predictors and the risk of retinopathy/nephropathy, facilitating the identification of hereditary factors of type 1 diabetes mellitus complications.

The literature reports significant associations between two polymorphic variants: rs3825932 in the *CTSH* gene and rs2292239 in the *ERBB3* gene, and the development and progression of diabetic retinopathy, with their effects being opposite. Thorsen et al. (2015) found that the rs3825932 (T>C) polymorphic variant in the *CTSH* gene is associated with a reduced risk of progression of proliferative diabetic retinopathy in patients with type 1 diabetes [17]. The analysis showed that carriers of the *T*/*T* genotype have a 5-fold reduced risk of developing DR compared to carriers of the *C*/*C* and *C*/*T* genotypes [17]. It has previously been established that the T allele of the rs3825932 variant is associated with reduced expression of the *CTSH* gene in lymphoid cells and pancreatic tissue. This may impact β-cell function and the progression of T1DM [16]. In another study using Mendelian randomization, variation in the *CTSH* gene was a risk factor for both diabetic retinopathy and diabetic maculopathy. The *CTSH* gene encodes the lysosomal enzyme cathepsinH, a cysteine protease that is involved in the processing and degradation of intracellular proteins, regulates immune responses (including in pancreatic islets), and maintains pancreatic β-cell homeostasis [36]. The *ERBB3* gene encodes the HER3 receptor (a receptor tyrosine kinase of the epidermal growth factor receptor family), which forms heterodimers with HER2/NEU (ERBB2) and activates the PI3K/AKT and MAPK signaling pathways, regulating cell proliferation, differentiation, and survival, and is involved in angiogenesis and vascular remodeling. In the retina, *ERBB3* is expressed in endothelial cells and glial elements, influencing blood-retinal barrier permeability, neovascularization, and neuroprotection [37]. To date, several genome-wide association studies have been conducted in various populations using different definitions of DR. However, none of the results have been consistently replicated, which is most likely due to insufficient sample size and inconsistent cross-sectional comparisons of the DR definitions used in earlier studies [38].

Since the key pathogenetic link in both T1D and its complications is chronic immune-inflammatory damage to the β-cells of the islets of Langerhans, it seems logical to include markers in genes that influence immune system function. In particular, the *TRIM31* gene, which encodes a protein involved in the regulation of innate immunity and inflammation through pathways associated with NF-κB and inflammasomes [39]. Its variant rs2523989 in the genome-wide study by Tomer et al. (2015) is associated with the risk of T1D [40]; however, its role in the development of microvascular complications remains unclear. The *CD226* gene encodes a costimulatory receptor on the surface of T cells and NK cells [41]. The polymorphic variant rs763361 (missense substitution) potentially affects the function of this receptor and is associated not only with type 1 diabetes, but also with other autoimmune pathologies [42]. Its role in lymphocyte adhesion to the endothelium may be key to the mechanisms of retinal and renal vascular injury. The *CLEC16A* gene is of particular interest. It is a strong susceptibility locus for type 1 diabetes, whose function is associated with the regulation of autophagy and antigen presentation, which are critical for cellular homeostasis. Gene variability may influence autophagy in endothelial cells and podocytes under the influence of hyperglycemia and may be involved in the central mechanisms of microvascular complications [43].

Of interest are groups of genes closely associated with progressive structural and functional damage to certain cell types and tissues that, due to their biochemical and physiological characteristics, are more vulnerable to the chronic effects of hyperglycemia and other metabolic disturbances associated with diabetes. The *LRP5* gene encodes a co-receptor in the WNT signaling pathway. In addition to its role in bone metabolism, this pathway plays a critical role in angiogenesis and maintaining the integrity of the blood-retinal barrier [44]. Mutations in *LRP5* are known to be associated with familial exudative vitreoretinopathy, which shares phenotypes with proliferative diabetic retinopathy. Therefore, variants of this gene may be associated with a risk of retinal vascular damage [45].

Our replication study identified polymorphic variants associated with the risk of developing type 1 diabetes mellitus and its microvascular complications. The rs2292239 polymorphism in the *ERBB3* gene demonstrated an association with an increased risk of developing type 1 diabetes in general (p_bonf_ = 0.001, OR = 2.02), while rs55703767 in the *COL4A3* gene exerted a protective effect on the risk of T1DM in general (*p* = 0.018, OR = 0.42). Notably, in the model that included these loci, male gender acted as a protective factor in the prognostic model (β = −1.09812). For diabetic nephropathy, rs9344715 in the *AKIRIN2* gene (*p* = 0.04, OR = 1.29) was a significant risk factor, while the rs77924615 variants in the *PDILT* gene (*p* = 0.013, OR = 1.59) and rs11864909 in the *PDILT* gene (*p* = 0.015, OR = 1.52) were associated with an increased risk of DR, as well as with a combined form of CKD and DR compared to individuals without complications.

### 4.1. ERBB3

The *ERBB3* gene encodes a membrane protein that is a member of the epidermal growth factor receptor (EGFR/ErbB) family of receptor tyrosine protein kinases. Overexpression of this gene has been detected in numerous cancers, including prostate, bladder, and breast cancers. Alternative transcriptional splice variants encoding different isoforms have been characterized. One isoform lacks the intermembrane region and is secreted extracellularly. This form modulates the activity of the membrane-bound form [46]. The gene is known to regulate cell survival, differentiation, and proliferation in various tissue types via PIK3/Akt. ERBB3 is thought to contribute to the pathogenesis of type 1 diabetes mellitus; it is highly expressed in monocytes and dendritic cells, influencing the functionality of antigen-presenting cells, autoimmunity, and cytokine-induced β-cell apoptosis [47]. Genome-wide association studies confirmed this by linking *ERBB3* gene variation with susceptibility to T1D, with the strongest association signal observed for the rs2292239 polymorphism in intron 7 of the gene. Furthermore, the gene is highly expressed in pancreatic β-cells. GTEx data indicate an eQTL effect of rs2292239 on *ERBB3* expression in the pancreas (*p* = 5.31 × 10^−12^) [48]. Interestingly, the A allele of the rs705708 polymorphism in the *ERBB3* gene is associated with a reduced risk of diabetic retinopathy and hypertension, as well as improved renal function in patients with type 1 diabetes [49].

A review of meta-analyses of the rs2292239 polymorphism in the *ERBB3* gene and its association with the risk of developing type 1 diabetes mellitus confirms a significant association of the *T* allele with an increased predisposition to the disease in the general population (OR = 1.292), as well as in subgroups of European and Asian descent [50]. These data are consistent with our results. The observed difference in the OR value (1.292 in the meta-analysis versus 2.02 in our cohort) may reflect population-specific effects, differences in study design, or consideration of additional factors such as a gender modifier (β = −1.09812 for males in our model). In the study by Lemos et al. (2018), the *T*/*T* genotype of the single-nucleotide polymorphism rs2292239 was associated with the risk of developing T1DM in the Brazilian, which is consistent with our results [48].

### 4.2. COL4A3

The *COL4A3* gene encodes a protein that is the *COL4A3* subunit of type IV collagen (collagen type IV class alpha-3 (the α-3 chain of collagen type IV)), the main structural component of the glomerular basement membrane (the glomerular basement membrane of the kidneys), consisting of six subunits. It is expressed primarily in the glomeruli of the kidneys, skeletal muscle, and lungs [51]. Defects in the *COL4A3* gene are associated with progressive kidney failure [52]. In addition, certain gene variants are associated with the development of Alport syndrome types 2 and 3 and familial benign hematuria [53,54,55]. An international genomic consortium funded by the Joint Diabetic Research Foundation (JDRF) has collected nearly 20000 samples from participants with type 1 diabetes, with and without kidney disease [11]. The authors identified 16 new loci associated with diabetic kidney disease with genome-wide significance. The strongest signal was concentrated in the protective missense coding variant rs55703767 of the *COL4A3* gene. Our data are fully consistent with the results of the study, since, as in our study, the minor allele of rs55703767 (Asp326Tyr) protects against several definitions of diabetic kidney disease, including albuminuria, and demonstrates a significant association with basement membrane width. Furthermore, carriers of the protective allele had a thinner basement membrane before the onset of any signs of kidney disease, and its effect depended on glycemia [11]. The interpretation and potential mechanism of the protective effect of the minor allele are presented in the article by Mirtani and coauthors, where the author suggested that the inherited presence of the minor allele (Asp326Tyr) in the *COL4A3* gene may lead to mild endoplasmic reticulum impairment and persistent activation of the cytoprotective (adaptive) branch of the unfolded protein response pathway in podocytes of patients with diabetes, acting as a form of “natural preconditioning” of the cells. Thus, this specific mutation may represent a “mild genetic challenge” for the podocyte, which is then prepared to withstand further endoplasmic reticulum stress caused by diabetes. This hypothesis is consistent with the fact that the effect of the *COL4A3* variant on CKD risk is enhanced by poor glycemic control [56].

### 4.3. AKIRIN2

*AKIRIN2* is a gene that encodes a molecular adapter that acts as a bridge between various multiprotein complexes and is involved in embryonic development, immunity, myogenesis, and brain development. Protein expression and activity are controlled by a complex interplay of transcriptional, post-transcriptional, and post-translational mechanisms, ensuring spatial and temporal regulation of cellular processes [57]. AKIRIN2 is known to regulate transcriptional control by recruiting the SWI/SNF complex (a family of ATP-dependent proteins that remodel chromatin by relocalizing nucleosomes to make DNA more accessible for gene transcription) in B lymphocytes [58].

In the GWAS database, the polymorphism rs9344715 was identified as a marker that affects glomerular filtration rate [22]; however, there are no other references in the world literature about the role of this variant in the risk of developing nephropathy. In our study, the minor allele and genotypes of this variant were significantly associated with the risk of diabetic nephropathy in type 1 diabetes, providing further support for the potentially important role of this marker. However, the precise mechanisms underlying the variant’s pathogenetic effect remain a subject for future research and replication.

### 4.4. PDILT and UMOD

The *PDILT* gene encodes a protein in the endoplasmic reticulum (ER) disulfide isomerase family, which catalyzes protein folding and thiol-disulfide exchange reactions. The adjacent *UMOD* gene encodes the protein uromodulin (also known as Tamm-Horsfall protein), which is synthesized in the kidneys, specifically in the loop of Henle, and helps reabsorb important substances, preventing their loss in the urine. Mutations in the *UMOD* gene are associated with rare inherited kidney diseases, including autosomal dominant tubulointerstitial kidney disease (ADTKD-UMOD) and medullary cystic kidney disease type 2 (MCKD2), characterized by progressive kidney failure and elevated uric acid levels [59]. Robinson-Cohen et al. published the results of a GWAS in 2023 to examine the association of genomic variation with glomerular filtration rate among individuals with established CKD of African and European descent, stratified by diabetes status. The findings identified two loci associated with CKD and confirmed the associations of rs77924615 (*PDILT*) and rs11864909 (*PDILT*) with glomerular filtration rate in individuals with diabetes [60].

The polymorphic locus rs77924615 of the *PDILT* gene is an intronic variant and, according to studies, modulates the activity of the *UMOD* gene enhancer, influencing the deterioration of kidney function in patients with the progression of immunoglobulin A (IgA) nephropathy by increasing the level of uromodulin both in the epithelium of the renal tubules and in the urine [61]. A GWAS study showed an association between the A allele and kidney stone disease and vitamin D levels [62]. Our study provides new data for the first time: an association of a minor gene variant with a reduced risk of retinopathy in type 1 diabetes, as well as a link between the rs11864909 variant and an increased risk of the combination of chronic kidney disease and diabetic retinopathy in type 1 diabetes mellitus.

The associations obtained in the present replication study are novel because they demonstrate, for the first time, a protective effect in the ophthalmic phenotype of T1DM, likely explained by the systemic effects of the *UMOD* and *PDILT* genes on vascular permeability and barrier function (similar to their role in preventing endothelial dysfunction in retinal vessels through inhibition of NF-κB-mediated inflammation and glycocalyx stabilization). The discrepancy with the associations observed for CKD may reflect a certain tissue specificity: in the kidneys, the *UMOD* gene enhances local fibrosis, whereas in the retina, its elevated levels (via the circulating pool) likely suppress hypoxia-induced neovascularization and oxidative stress. However, further studies are needed to confirm these hypotheses, including investigation of the differential expression of these genes in retinal tissues and functional models with targeted gene modifications. Mechanistic refinements are needed, integrating sex and ethnic modifiers, to expand the predictive value of these variants in models of T1DM complications.

## 5. Conclusions

This replication study confirmed the contribution of the rs2292239 polymorphism in the *ERBB3* gene to type 1 diabetes risk, with the association remaining significant after strict Bonferroni correction. Model-specific associations of *PDILT* variants rs77924615 and rs11864909 with diabetic retinopathy remained significant after Bonferroni correction for the tested inheritance models. Additionally, the rs11864909 variant was associated with the combined retinopathy-CKD phenotype, with the protective effect of the T/T genotype surviving multiple-testing correction (p_bonf_ = 0.043). Nominal signals were observed for rs55703767 (*COL4A3*) and rs9344715 (*AKIRIN2*), though these did not survive correction for multiple comparisons. Male sex emerged as an independent protective factor in the T1DM prediction model. While the *ERBB3* and *PDILT* findings highlight potential genetic contributors to T1DM susceptibility and microvascular complications, all associations should be interpreted as hypothesis-generating and require validation in independent, well-powered cohorts.

### Limitations of the Study

A limitation of our study is the small sample size and the limited number of polymorphic variants examined, as well as the inability to perform a formal correction for population stratification using principal component analysis due to the small number of genotyped SNPs. Furthermore, the obtained genetic associations require further validation in prospective studies with full consideration of various modifiable risk factors. However, the primary objective of the study was to replicate the results of GWAS in a population of Russian men and women with type 1 diabetes. The absence of longitudinal HbA1c data and detailed insulin therapy records is a limitation of this study. The study design, focused on replicating known associations, did not involve functional analysis of the identified significant variants, leaving the question of their specific molecular mechanisms open. It should also be noted that clinical stratification of patients by the stages of diabetic retinopathy and nephropathy for a more detailed analysis was limited by the sample size. Nevertheless, in the context of the primary objective—independent replication of large-scale GWAS findings in a specific cohort of Russian patients with type 1 diabetes—these restrictions are acceptable. The obtained results, even taking these limitations into account, are valuable for validating and refining genetic markers of microvascular risk in this population and provide a basis for subsequent studies in larger cohorts using whole-genome analysis and functional genomics approaches. The results should be viewed as suggestive and require confirmation in independent, well-designed cohorts.

## Figures and Tables

**Table 1:** Characteristics of the study sample.

Sample	Men	Female	Total Number
N (quantity)	228	390	618
Type 1 diabetes mellitus	172	350	522
Control group (N)	56	40	96
Characteristic	Men	Female	Total number
Height (cm)	173 ± 8.22	163.98 ± 7.79	167.32 ± 9.07
Weight (kg)	73.96 ± 13.91	65.46 ± 14.27	68.6 ± 14.72
Chronic kidney disease	81	160	241
Diabetic retinopathy	141	292	433
Mean age ± standard deviation	46.53 ± 13.08	46.57 ± 12.39	46.56 ± 12.65
Body mass index (BMI)	24.68 ± 3.80	24.33 ± 4.90	24.46 ± 4.53
Onset of T1DM (median ± standard deviation)	12 ± 9.00	11 ± 8.75	11 ± 8.30

**Table 2:** Characteristics of polymorphic variants included in the replication study.

SNP	Gene	Justification	Biological Role	Reference
rs9344715	*AKIRIN2*	Associated with glomerular filtration rate (*p* = 8 × 10^−11^).	A nuclear protein that controls the transport of 26S proteasomes into the cell nucleus.	[22]
rs55703767	*COL4A3*	Associated with diabetic nephropathy (*p* = 5 × 10^−12^).	Collagen type IV alpha-3 is a key component of the glomerular basement membranes of the kidneys.	[11]
rs4293393	*UMOD*	Associated with chronic kidney disease (*p* = 4 × 10^−10^).	Uromodulin modulates ion transport and inhibits stone formation.	[23]
rs11864909	*PDILT*	Associated with glomerular filtration rate (*p* = 2 × 10^−12^).	The gene encodes a protein of the disulfide isomerase (PDI) family, proteins of the endoplasmic reticulum (ER) that catalyze thiol-disulfide exchange reactions.	[24]
rs77924615		Associated with glomerular filtration rate (*p* = 3 × 10^−259^).		[25]
rs118124843	*VARS2*	Associated with microalbuminuria in type 1 diabetes (*p* = 4 × 10^−8^).	Valyl-tRNA synthetase 2 is responsible for the addition of valine to tRNA for protein synthesis within the mitochondria.	[11]
rs12917707	*UMOD*	Associated with chronic kidney disease (*p* = 2 × 10^−12^).	Uromodulin (see above).	[26]
rs73885319	*APOL1*	Associated with chronic kidney disease (*p* = 2 × 10^−9^).	Apolipoprotein L1 is involved in the immune response and apoptosis, and gene variants are associated with an increased risk of kidney failure.	[27]
rs183937294	*PLEKHA7*	Associated with albuminuria in T1DM (*p* = 2 × 10^−8^).	Involved in maintaining the integrity of the epithelial layer.	[11]
rs9622363	*APOL1*	Associated with chronic kidney disease (*p* = 2 × 10^−46^).	Apolipoprotein L1 (see above).	[28]
rs2523989	*TRIM31*	Associated with type 1 diabetes (*p* = 2 × 10^−8^)	The E3 ubiquitin ligase protein of the TRIM family is involved in ubiquitination, directing the degradation or stabilization of proteins.	[18]
rs3825932	*CTSH*	Associated with type 1 diabetes (*p* = 3 × 10^−15^)	Cathepsin H is a lysosomal cysteine protease involved in protein degradation and processing.	[29]
rs763361	*CD226*	Associated with type 1 diabetes (*p* = 1 × 10^−8^)	CD226 is expressed on the surface of NK cells, CD8+ T cells, and some CD4+ T cells.	[19]
rs12708716	*CLEC16A*	Associated with type 1 diabetes (*p* = 3 × 10^−18^)	C-lectin-like domain containing 16A, a protein that regulates endosomal trafficking and autophagy in immune cells.	[19]
rs2292239	*ERBB3*	Associated with type 1 diabetes (*p* = 2 × 10^−20^)	A membrane protein of the epidermal growth factor receptor family.	[19]
rs4948088	*COBL*	Associated with type 1 diabetes (*p* = 4 × 10^−8^)	Cordactin-like protein; involved in actin polymerization and formation of cell processes.	[30]
rs72831309	*TENM2*	Associated with chronic kidney disease (*p* = 1 × 10^−8^)	A protein that mediates cell adhesion molecule binding activity and signaling receptor binding efficiency.	[12]

**Table 3:** Characteristics of the studied polymorphic variants.

No.	Polymorphic Variants		Gene	Chromosomal Localization
1	rs9344715	n.87738792A>G	*AKIRIN2*	6q15
2	rs55703767	c.976G>A	*COL4A3*	2q36.3
3	rs4293393	c.-680T>C	*UMOD*	16p12.3
4	rs11864909	c.203-4666G>A	*PDILT*	16p12.3
5	rs77924615	c.409+3635C>T	*PDILT*	16p12.3
6	rs118124843	c.1075-70C>T	*VARS2*	6p21.33
7	rs12917707	c.-3782C>A	*UMOD*	16p12.3
8	rs73885319	c.1024A>G	*APOL1*	22q12.3
9	rs183937294	c.222-45117A>T	*PLEKHA7*	11p15.1
10	rs72831309	c.502+175752G>A	*TENM2*	5q34
11	rs9622363	c.188-1087A>G	*APOL1*	22q12.3
12	rs2523989	g.30110498C>A	*TRIM31*	6p22.1
13	rs3825932	g.78943104T>C	*CTSH*	15q25.1
14	*rs763361*	g.67531642T>A	*CD226*	18q22.2
15	rs12708716	g.146529A>G	*CLEC16A*	16p13.13
16	rs2292239	c.875-147T>G	*ERBB3*	12q13.2
17	rs4948088	g.51027194A>C	*COBL*	7p12.1

**Table 4:** Results of the analysis of the control sample for compliance with the Hardy–Weinberg equilibrium.

Gene	Loci	H_obs_	H_pred_	HW_pval_
*COL4A3*	rs55703767	0.333	0.404	0.083
*TENM2*	rs72831309	0.042	0.041	1.000
*TRIM31*	rs2523989	0.219	0.227	0.655
*VARS2*	rs118124843	0.042	0.041	1.000
*AKIRIN2*	rs9344715	0.531	0.499	0.682
*COBL*	rs4948088	0.083	0.080	1.000
*PLEKHA7*	rs183937294	0.010	0.010	1.000
*ERBB3*	rs2292239	0.354	0.364	0.781
*CTSH*	rs3825932	0.438	0.458	0.659
*CLEC16A*	rs12708716	0.469	0.426	0.471
*UMOD*	rs4293393	0.219	0.227	0.655
*UMOD*	rs12917707	0.219	0.227	0.655
*PDILT*	rs77924615	0.354	0.318	0.349
*PDILT*	rs11864909	0.417	0.422	1.000
*CD226*	rs763361	0.469	0.476	1.000
*APOL1*	rs9622363	0.010	0.010	1.000
*APOL1*	rs73885319	0.000	0.000	1.000

Notes: H_obs_—observed heterozygosity; H_pred_—expected heterozygosity; HW_pval_—*p*-level significance indicator of deviation from HD equilibrium.

**Table 5:** Results of the search for associations of polymorphic variants with type 1 diabetes mellitus in general.

Polymorphic Variant	Risk Allele	Allele Frequency in T1DM	Allele Frequency in the Control Group	χ^2^	*p*-Level	OR	L95	U95
rs55703767	*G*	0.771	0.719	2.455	0.117	1.32	0.932	1.863
rs72831309	*A*	0.024	0.021	0.069	0.793	1.15	0.397	3.351
rs2523989	*T*	0.146	0.130	0.313	0.576	1.14	0.723	1.793
rs118124843	*T*	0.000	0.021	*NA*	*NA*	*NA*	*NA*	*NA*
rs9344715	*A*	0.489	0.474	0.137	0.711	1.06	0.779	1.443
rs4948088	*C*	0.972	0.958	1.077	0.299	1.52	0.685	3.381
rs183937294	*G*	0.000	0.005	*NA*	*NA*	*NA*	*NA*	*NA*
rs2292239	*T*	0.389	0.240	15.590	7.89 × 10^−5^	2.02	1.418	2.878
rs3825932	*C*	0.694	0.646	1.710	0.191	1.24	0.898	1.715
rs12708716	*G*	0.314	0.307	0.036	0.850	1.03	0.740	1.441
rs4293393	*G*	0.178	0.130	2.634	0.105	1.45	0.924	2.269
rs12917707	*T*	0.170	0.130	1.835	0.176	1.36	0.869	2.139
rs77924615	*A*	0.212	0.198	0.186	0.667	1.09	0.741	1.599
rs11864909	*C*	0.705	0.698	0.039	0.844	1.03	0.740	1.447
rs763361	*T*	0.422	0.391	0.634	0.426	1.14	0.830	1.557
rs9622363	*G*	0.000	0.005	*NA*	*NA*	*NA*	*NA*	*NA*
rs73885319	*A*	1.000	1.000	*NA*	*NA*	*NA*	*NA*	*NA*

Notes: NA (Not Available)—indicates that it is impossible to calculate statistical indicators (*p*-value, OR, SE) for these genetic variants. The main reasons are the monomorphic status of the locus in the studied sample.

**Table 6:** Distribution of rs2292239 genotype frequencies in the group of patients with type 1 diabetes and the control group.

rs2292239	N	Number of Genotypes			Genotype Frequencies			Association
Genotypes	618	*T*/*T*	*T*/*G*	*G*/*G*	*T*/*T*	*T*/*G*	*G*/*G*	*T*/*T* − (*T*/*G* + *G*/*G*) χ^2^ = 5.58 OR = 2.71 *p* = 0.018
Type 1 diabetes mellitus	522	80	246	196	0.153	0.471	0.375	
Control group	96	6	34	56	0.063	0.354	0.583	

**Table 7:** Logistic regression coefficients for the final T1DM risk prediction model with sex factor.

Predictor	β	SE	95% CI (β)	*p*-Value	OR	95% CI (OR)
Intercept	[0.412]	[0.22]	[−0.02; 0.84]	[0.061]	–	–
rs55703767 (T/T vs. ref)	−1.271	[0.35]	[−1.96; −0.58]	[0.0003]	0.28	[0.14; 0.56]
rs2292239 (G/T vs. ref)	0.688	[0.28]	[0.14; 1.24]	[0.014]	1.99	[1.15; 3.45]
rs2292239 (T/T vs. ref)	1.364	[0.41]	[0.56; 2.17]	[0.001]	3.91	[1.75; 8.76]
Sex (Male vs. Female)	−1.098	[0.30]	[−1.69; −0.51]	[0.0002]	0.33	[0.18; 0.60]

**Table 8:** Results of the search for associations of polymorphic variants with diabetic nephropathy.

Polymorphic Variant	Risk Allele	Allele Frequency in CKD	Allele Frequency in the T1DM Group Without CKD	χ^2^	*p*-Level	OR	L95	U95
rs55703767	*G*	0.780	0.763	0.412	0.521	1.10	0.823	1.470
rs72831309	*G*	0.981	0.972	1.066	0.302	1.54	0.674	3.517
rs2523989	*C*	0.869	0.842	1.596	0.207	1.25	0.883	1.773
rs118124843	*T*	0.000	0.000	*NA*	*NA*	*NA*	*NA*	*NA*
rs9344715	*A*	0.523	0.459	4.220	0.040	1.29	1.012	1.648
rs4948088	*A*	0.029	0.027	0.053	0.817	1.09	0.521	2.284
rs183937294	*G*	0.000	0.000	*NA*	*NA*	*NA*	*NA*	*NA*
rs2292239	*G*	0.616	0.607	0.097	0.756	1.04	0.811	1.336
rs3825932	*C*	0.699	0.689	0.136	0.712	1.05	0.807	1.369
rs12708716	*A*	0.691	0.682	0.106	0.745	1.05	0.804	1.358
rs4293393	*G*	0.183	0.174	0.119	0.730	1.06	0.770	1.453
rs12917707	*T*	0.172	0.167	0.045	0.832	1.04	0.749	1.432
rs77924615	*A*	0.216	0.208	0.089	0.765	1.05	0.777	1.409
rs11864909	*C*	0.712	0.699	0.190	0.663	1.06	0.812	1.386
rs763361	*T*	0.430	0.415	0.235	0.628	1.06	0.831	1.360
rs9622363	*G*	0.000	0.000	*NA*	*NA*	*NA*	*NA*	*NA*
rs73885319	*A*	1.000	1.000	*NA*	*NA*	*NA*	*NA*	*NA*

**Table 9:** Distribution of rs9344715 genotype frequencies in the group of patients with T1DM with and without nephropathy.

rs9344715	N	Number of Genotypes			Genotype Frequencies			Association
Genotypes	522	*A*/*A*	*A*/*G*	*G*/*G*	*A*/*A*	*A*/*G*	*G*/*G*	*A*/*A* − (*A*/*G* + *G*/*G*) χ^2^ = 5.310 OR = 1.65 *p* = 0.021
CKD in type 1 diabetes	241	60	132	49	0.249	0.548	0.203	
Type 1 diabetes without CKD	281	47	164	70	0.167	0.584	0.249	

**Table 10:** Results of the search for associations of polymorphic variants with diabetic retinopathy.

Polymorphic Variant	Risk Allele	Allele Frequency in DR	Allele Frequency in the T1DM Group Without DR	χ^2^	*p*-Level	OR	L95	U95
rs55703767	*T*	0.232	0.214	0.290	0.590	1.11	0.753	1.648
rs72831309	*G*	0.977	0.972	0.158	0.691	1.22	0.453	3.302
rs2523989	*C*	0.858	0.837	0.518	0.472	1.18	0.756	1.828
rs118124843	*T*	0.000	0.000	*NA*	*NA*	*NA*	*NA*	*NA*
rs9344715	*A*	0.494	0.461	0.665	0.415	1.14	0.828	1.581
rs4948088	*C*	0.972	0.972	0.001	0.978	1.01	0.382	2.694
rs183937294	*G*	0.000	0.000	*NA*	*NA*	*NA*	*NA*	*NA*
rs2292239	*G*	0.617	0.584	0.651	0.420	1.14	0.824	1.589
rs3825932	*T*	0.311	0.287	0.404	0.525	1.12	0.787	1.601
rs12708716	*G*	0.320	0.287	0.762	0.383	1.17	0.821	1.670
rs4293393	*A*	0.826	0.803	0.500	0.480	1.16	0.770	1.745
rs12917707	*G*	0.834	0.815	0.383	0.536	1.14	0.751	1.734
rs77924615	*G*	0.803	0.719	6.160	0.013	1.59	1.100	2.292
rs11864909	*C*	0.721	0.629	5.923	0.015	1.52	1.083	2.131
rs763361	*T*	0.430	0.382	1.368	0.242	1.22	0.875	1.696
rs9622363	*G*	0.000	0.000	*NA*	*NA*	*NA*	*NA*	*NA*
rs73885319	*A*	1.000	1.000	*NA*	*NA*	*NA*	*NA*	*NA*

**Table 11:** Distribution of rs77924615 genotype frequencies in the group of patients with T1DM with retinopathy and T1DM without retinopathy.

rs77924615	N	Number of Genotypes			Genotype Frequencies			Association
Genotypes	522	*A*/*A*	*A*/*G*	*G*/*G*	*A*/*A*	*A*/*G*	*G*/*G*	*A*/*A* − (*G*/*G*) χ^2^ = 5.815 OR = 0.57 *p* = 0.015
T1DM with retinopathy	433	25	121	287	0.058	0.279	0.663	
T1DM without retinopathy	89	8	34	47	0.090	0.382	0.528	

**Table 12:** Distribution of rs11864909 genotype frequencies in the group of patients with T1DM with retinopathy and T1DM without retinopathy.

rs11864909	N	Number of Genotypes			Genotype Frequencies			Association
Genotypes	522	*T*/*T*	*T*/*C*	*C*/*C*	*T*/*T*	*T*/*C*	*C*/*C*	*T*/*T* − (*T*/*C* + *C*/*C*) χ^2^ = 8.52 OR = 0.41 *p* = 0.0035
T1DM with retinopathy	433	41	160	232	0.095	0.370	0.536	
T1DM without retinopathy	89	18	30	41	0.202	0.337	0.461	

**Table 13:** Results of the search for associations of polymorphic variants with a combined form of complications.

Polymorphic Variant	Risk Allele	Allele Frequency in CKD with DR	Allele Frequency in the Group of Type 1 Diabetes Without CKD and DR	χ^2^	*p*-Level	OR	L95	U95
rs55703767	*T*	0.224	0.225	0.001	0.982	1.00	0.647	1.530
rs72831309	*G*	0.981	0.969	0.762	0.383	1.63	0.538	4.940
rs2523989	*C*	0.873	0.844	0.865	0.353	1.27	0.766	2.110
rs118124843	*T*	0.000	0.000	*NA*	*NA*	*NA*	*NA*	*NA*
rs9344715	*A*	0.524	0.456	2.166	0.141	1.31	0.914	1.879
rs4948088	*C*	0.970	0.969	0.005	0.946	1.04	0.368	2.926
rs183937294	*G*	0.000	0.000	*NA*	*NA*	*NA*	*NA*	*NA*
rs2292239	*G*	0.612	0.569	0.931	0.335	1.20	0.831	1.722
rs3825932	*T*	0.306	0.300	0.020	0.886	1.03	0.696	1.522
rs12708716	*G*	0.313	0.294	0.196	0.658	1.09	0.738	1.619
rs4293393	*A*	0.825	0.825	0.000	0.990	1.00	0.625	1.610
rs12917707	*G*	0.834	0.831	0.007	0.935	1.02	0.631	1.650
rs77924615	*G*	0.789	0.725	2.754	0.097	1.42	0.938	2.140
rs11864909	*C*	0.713	0.625	4.348	0.037	1.49	1.023	2.179
rs763361	*T*	0.431	0.381	1.212	0.271	1.23	0.851	1.777
rs9622363	*G*	0.000	0.000	*NA*	*NA*	*NA*	*NA*	*NA*
rs73885319	*A*	1.000	1.000	*NA*	*NA*	*NA*	*NA*	*NA*

**Table 14:** Distribution of rs11864909 genotype frequencies in the group of patients with type 1 diabetes with and without associated complications.

rs11864909	N	Number of Genotypes			Genotype Frequencies			Association
Genotypes	312	*T*/*T*	*T*/*C*	*C*/*C*	*T*/*T*	*T*/*C*	*C*/*C*	*T*/*T* − (*T*/*C* + *C*/*C*) χ^2^ = 6.01 OR = 0.44 *p* = 0.014
T1DM with CKD and DR	232	26	81	125	0.112	0.349	0.539	
T1DM without complications	80	17	26	37	0.213	0.325	0.463	

**Table 15:** Estimates of the probability of a variant’s influence on the regulation of gene expression according to the Regulome DB database.

Loci	Transcription Factors and Their Binding Sites		RegulomeDB Rank
rs2292239	Chip data	CTCF, POLR2A, RBFOX2, CEBPA	1f—eQTL/caQTL + TF binding/chromatin accessibility peak
rs77924615	Chip data	NFIC, ZNF24, NFIA, HES4, CEBPA, SALL1, TBX2, NFIX, NFIB, HNF1A, NFIB, NFIC, HNF1A, LCORL	1a—eQTL/caQTL + TF binding + matched TF motif + matched Footprint + chromatin accessibility peak
rs9344715	Chip data	ATF7, MTA2, IKZF1, DPF2, MLLT1, TBX21	1f—eQTL/caQTL + TF binding/chromatin accessibility peak

**Table 16:** Estimates of the probability of a variant’s influence on the regulation of gene expression according to the MoBiDiC MPA prioritization algorithm.

Loci	CADD Phred	FATHMM Converted Rankscore	MPA Score
rs55703767	19.99	0.938	2

Notes: CADD Phred—log-transformed CADD pathogenicity scores; FATHMM converted rankscore—normalized FATHMM pathogenicity score; MPA score—an aggregated pathogenicity score developed for ranking single-nucleotide variants, the higher the score, the higher the priority of the variant for further clinical evaluation.

## Data Availability

The data from this study can be obtained from the corresponding author upon making a reasonable request if there are no privacy or ethical issues.
